# Neuroendocrine and psychosocial triggers of severe psychiatric onset in women: a systematic review with domain-specific quantitative and narrative synthesis

**DOI:** 10.3389/fpsyt.2026.1889528

**Published:** 2026-07-29

**Authors:** Senay Kilincel, Furkan Bulut, Esma Akpinar Aslan, Oguzhan Kilincel

**Affiliations:** 1 Department of Child and Adolescent Psychiatry, Faculty of Medicine, Istanbul Aydın University, Istanbul, Türkiye; 2 Sakarya Child and Adolescent Psychiatry Institute, Sakarya, Türkiye; 3 Department of Psychiatry, Faculty of Medicine, Tokat Gaziosmanpaşa University, Tokat, Türkiye; 4 Department of Child Development, İstanbul Gelisim University Faculty of Health Sciences, Istanbul, Türkiye

**Keywords:** first-episode mania, intimate partner violence, neuroendocrine transition, perimenopause, postpartum psychosis, reproductive mental health

## Abstract

**Aim:**

Severe psychiatric onsets in women show pronounced clustering around specific reproductive life stages. This study aimed to systematically evaluate (i) the association between the perimenopausal transition and first-episode mania and (ii) the relationship between intimate partner violence (IPV) and postpartum psychosis (PPP), integrating neuroendocrine and psychosocial perspectives across the female life course.

**Methods:**

A systematic review with domain-specific quantitative and narrative synthesis was conducted in accordance with PRISMA 2020 guidelines. PubMed/MEDLINE, Embase, Web of Science, Scopus, PsycINFO, and Cochrane CENTRAL were searched from inception to December 2024. Observational studies reporting first-onset mania during perimenopause or PPP in relation to IPV exposure were included. Random-effects models were planned where quantitative pooling was feasible. Risk of bias was assessed using the Newcastle–Ottawa Scale, and certainty of evidence was evaluated with the GRADE framework.

**Results:**

Three studies met criteria for quantitative synthesis and seven for contextual narrative synthesis. A large population-based cohort demonstrated a significantly increased risk of first-onset mania during the perimenopausal transition (RR = 2.1, 95% CI: 1.30–3.52; low-certainty evidence). In contrast, direct evidence linking IPV to PPP was sparse and heterogeneous, precluding pooled effect estimation (very low-certainty evidence). Contextual evidence from systematic reviews, meta-analyses, and cohort studies consistently associated IPV with adverse perinatal mental health outcomes, particularly postpartum depression and anxiety (moderate-certainty evidence).

**Discussion:**

The findings support perimenopause as a clinically meaningful risk window for first-onset mania and highlight a critical evidence gap regarding IPV as a potential trigger for PPP. Although direct quantitative evidence is limited, biological plausibility and consistent contextual findings suggest that intense psychosocial stressors such as IPV may amplify postpartum psychiatric vulnerability in susceptible individuals. Future large-scale, well-phenotyped perinatal cohorts are needed to directly quantify this association.

## Highlights

- This systematic review investigated two reproductive life-stage–related psychiatric conditions in women: first-episode mania during perimenopause and postpartum psychosis associated with intimate partner violence (IPV).
- The findings suggest that the perimenopausal transition may represent a clinically significant risk window for first-onset mania, with population-based evidence demonstrating an approximately twofold increased risk.
- Direct evidence linking IPV to postpartum psychosis remains limited and heterogeneous; however, contextual evidence consistently associates IPV with adverse perinatal mental health outcomes, including postpartum depression and anxiety.
- The study highlights the potential interaction between neuroendocrine transitions, psychosocial stressors, circadian disruption, and immune dysregulation in the pathogenesis of severe psychiatric onset in women.
- The review emphasizes the need for trauma-informed perinatal psychiatric care and greater clinical awareness of reproductive-transition–related psychiatric vulnerability.
- The manuscript identifies substantial evidence gaps and calls for large prospective cohorts with standardized assessment of IPV exposure, reproductive staging, and psychiatric outcomes.

## Introduction

Severe psychiatric onsets in women show striking clustering around specific reproductive life stages, suggesting that biological transitions and psychosocial exposures may interact to amplify risk in vulnerable individuals. Two clinically important—but typically siloed—phenomena are first-episode mania emerging during the perimenopausal transition and postpartum psychosis (PPP) occurring after childbirth, a psychiatric emergency associated with high morbidity and the need for rapid recognition and treatment. Recent population-scale evidence has strengthened the argument that perimenopause is not merely a period of nonspecific distress but may represent a discrete window of heightened risk for new-onset mania, with risk estimates exceeding those observed for several other diagnostic outcomes (1). In a large UK Biobank analysis, perimenopause was associated with an increased incidence of first-onset mania, with the largest perimenopausal effect reported for mania compared to other diagnostic categories (2).

In this review, first-onset mania refers to the first lifetime clinically diagnosed manic episode occurring in women without a previous history of mania or bipolar disorder, ascertained using standardized diagnostic criteria or validated health record–based diagnoses. Mechanistically, perimenopause is characterized by pronounced variability and overall decline in ovarian steroids, particularly estradiol, which modulate neural systems implicated in affect regulation, reward processing, sleep–wake stability, and stress responsivity (3). These neurobiological domains overlap with core pathophysiological models of bipolar-spectrum disorders. Clinical syntheses have emphasized that the menopausal transition may precipitate first-onset mood episodes through hormone-sensitive changes in neurotransmission and neurocircuit function, while also interacting with sleep disruption, vasomotor symptoms, and psychosocial stressors common in midlife (4). Although individual studies and narrative reviews increasingly describe this pattern, the evidence has not been quantitatively consolidated to estimate the magnitude of risk for first-episode mania across perimenopause or to identify moderators such as age, menopausal staging methods, baseline psychiatric history, and ascertainment strategy.

PPP is an acute psychiatric emergency characterized by hallucinations, delusions, severe mood symptoms, and cognitive disorganization, typically occurring within the first days to weeks after childbirth (5). In the present review, PPP diagnoses were based on clinician-confirmed DSM- or ICD-based criteria or equivalent validated diagnostic procedures reported in the original studies. PPP represents a second high-risk context of severe onset, typically emerging within weeks after delivery and strongly associated with bipolar-spectrum vulnerability. Contemporary reviews underscore that PPP is rare but severe, with rapid symptom escalation and substantial risk for self-harm and harm to the infant if untreated (6). The postpartum period is also marked by abrupt endocrine shifts (including the withdrawal of pregnancy-level estradiol and progesterone), immune rebound, and major changes in sleep and circadian rhythms—factors plausibly capable of precipitating psychosis in predisposed women. Beyond biological triggers, however, psychosocial trauma has increasingly been discussed as a potential amplifier of postpartum psychiatric outcomes. A systematic review specifically examining adverse life events and PPP concluded that the available evidence was limited and heterogeneous, and highlighted the need for clearer operationalization of stressor exposure and better powered studies (7).

Within psychosocial exposures, intimate partner violence (IPV) is especially relevant due to its high global prevalence and its established association with a range of perinatal mental health outcomes (8). The World Health Organization estimates that roughly 30% of women worldwide experience physical and/or sexual violence by an intimate partner or non-partner across their lifetime, reflecting a major public health burden (9). Perinatal IPV has been linked to elevated risk of depression, anxiety, and perinatal suicidality, and broader syntheses in perinatal populations consistently report associations between IPV exposure and adverse mental health outcomes (10–12). Despite the plausibility of IPV contributing to PPP via trauma-related pathways—such as heightened HPA-axis activation, sleep disruption, and immune dysregulation—IPV has rarely been evaluated as a risk factor for postpartum psychosis specifically, and no quantitative synthesis has established the magnitude of association or explored sources of heterogeneity (e.g., timing of IPV exposure, severity/type of violence, comorbid PTSD, parity, or bipolar history).

Integrating these two domains—perimenopausal first-onset mania and IPV-associated PPP—offers a conceptual advantage. Both conditions may reflect threshold crossings in severe psychopathology during periods when endocrine and stress systems are highly labile, and both may be shaped by moderators spanning biology (hormonal dynamics, sleep/circadian disruption, immune activation) and context (trauma exposure, socioeconomic adversity, healthcare access). Emerging clinical observations have also noted that some women experience reproductive-transition–linked clustering of severe mood and psychotic episodes across life stages, including postpartum and menopause-associated episodes (13). Rather than addressing two unrelated clinical questions, this review was intentionally designed around a single overarching conceptual framework: severe psychiatric onset occurring during female reproductive transition states. Perimenopausal first-onset mania and postpartum psychosis represent two clinically distinct manifestations arising at different stages of the reproductive life course, yet both occur during periods characterized by profound neuroendocrine instability, circadian disruption, immune modulation, and heightened vulnerability to psychosocial stressors. Accordingly, these conditions were examined as complementary models of reproductive transition–related psychiatric vulnerability rather than as independent disease entities. A common systematic review methodology was applied across both predefined evidence domains, whereas quantitative synthesis was conducted separately within each domain whenever sufficient homogeneous data were available. Thus, integration was conceptual rather than statistical, aiming to develop a unified life-course framework for understanding severe psychiatric onset in women. This approach improves interpretability by enabling comparison of shared biological mechanisms across reproductive transition stages while preserving methodological independence for each evidence domain.

Accordingly, the present work proposes a systematic review and meta-analysis with two objectives. First, we aim to quantify the association between perimenopause and first-onset mania, synthesizing available epidemiologic evidence and evaluating moderators related to menopausal staging, age, ascertainment method, and baseline risk profiles. Second, we aim to quantify the association between IPV exposure and postpartum psychosis, integrating data across study designs and settings and exploring heterogeneity by IPV definition (physical, sexual, psychological), exposure timing (pregnancy vs postpartum), and clinical risk factors (parity, bipolar disorder history). By synthesizing these literatures in parallel, this study seeks to advance a more integrated, sex- and stage-informed understanding of severe psychiatric onset, and to identify actionable points for risk detection and prevention. Across the included studies, diagnostic ascertainment was based on clinician-confirmed diagnoses using DSM- or ICD-based criteria or validated health record–based diagnostic coding, depending on the study design.

## Methods

### Study design and reporting standards

This review was not prospectively registered in PROSPERO or any other systematic review registry. Nevertheless, the review protocol, eligibility criteria, and analytical plan were predefined before study selection, data extraction, and evidence synthesis, and the review was conducted in accordance with the PRISMA 2020 guidelines (14). This review was designed as a single systematic review addressing one overarching research question: how reproductive transition states contribute to severe psychiatric onset in women. Within this framework, two predefined evidence domains were evaluated in parallel: (i) perimenopausal first-onset mania and (ii) postpartum psychosis in relation to intimate partner violence. A common systematic review methodology was applied across both domains, including identical search procedures, study selection, data extraction, risk-of-bias assessment, and certainty evaluation. Quantitative synthesis was performed separately within each domain whenever sufficient homogeneous evidence was available because the underlying clinical questions, outcome definitions, and available data differed substantially. Consequently, cross-domain integration was limited to conceptual interpretation rather than statistical pooling, allowing comparison of shared neuroendocrine and psychosocial mechanisms while preserving the methodological independence of each evidence domain.

### Literature search strategy

A comprehensive literature search was performed in PubMed/MEDLINE, Embase, Web of Science, Scopus, PsycINFO, and the Cochrane CENTRAL database from inception to December 2024. Gray literature sources, including ClinicalTrials.gov and reference lists of relevant reviews, were also screened. Search strategies combined controlled vocabulary (e.g., MeSH, Emtree) and free-text terms related to perimenopause, first-episode mania, postpartum psychosis, and intimate partner violence. Search strategies were adapted for each database using controlled vocabulary and free-text terms. Only studies published in English were included. No translation of non-English publications was performed.

### Research question (PECOS framework)

The review question was structured according to the PECOS framework. The population comprised women undergoing the perimenopausal transition or the pregnancy/postpartum period. The exposures of interest were the perimenopausal transition and intimate partner violence. Comparators included women outside the perimenopausal transition or women without documented IPV exposure, where applicable. The primary outcomes were first-onset mania and clinically diagnosed postpartum psychosis. Eligible study designs included observational studies (cohort, case–control, and cross-sectional studies), whereas reviews and case series were considered only as contextual evidence.

### Eligibility criteria

#### Perimenopausal first-episode mania

Studies were eligible if they (1) included women in the perimenopausal or menopausal transition stage, (2) reported first-onset mania or bipolar-spectrum manic episodes, and (3) provided quantitative risk estimates or sufficient data to derive effect sizes. Studies focusing exclusively on recurrent bipolar disorder or mixed-sex samples without sex-stratified data were excluded.

#### Intimate partner violence and postpartum psychosis

Studies were included if they (1) examined women in the perinatal or postpartum period, (2) reported clinically diagnosed postpartum psychosis, and (3) assessed exposure to IPV (physical, sexual, psychological, or combined). Studies evaluating only postpartum depression or anxiety without psychotic outcomes were excluded.

#### Data extraction

Two reviewers independently screened titles, abstracts, and full texts. Discrepancies were resolved by consensus. Extracted data included study design, country, sample size, participant characteristics, diagnostic criteria, menopausal or postpartum timing, IPV definition and timing, effect estimates (odds ratios, hazard ratios, or incidence rate ratios), and covariates used in adjusted analyses. Information regarding diagnostic ascertainment methods, including DSM- or ICD-based diagnostic criteria, clinician-confirmed diagnoses, health record–based ascertainment, and the reported postpartum onset window, was extracted from each eligible study to evaluate diagnostic heterogeneity.

#### Risk of bias assessment

Risk of bias was assessed using the Newcastle–Ottawa Scale (NOS) for observational studies (15). Domains evaluated included selection of participants, comparability of study groups, and outcome assessment. Studies were categorized as having low, moderate, or high risk of bias based on standard NOS thresholds.

#### Data synthesis and statistical analysis

Quantitative synthesis was planned only when at least two studies were sufficiently homogeneous with respect to population, exposure, outcome definition, study design, and effect measures to permit meaningful statistical pooling. Random-effects models were prespecified because clinical and methodological heterogeneity was anticipated across observational studies. Heterogeneity was intended to be assessed using Cochran’s Q test and the I² statistic. Planned subgroup analyses included menopausal staging methods, age groups, IPV subtype, and timing of exposure, whereas sensitivity analyses were intended to evaluate the robustness of pooled estimates by excluding studies at higher risk of bias. However, after study selection, only one study met the predefined criteria for quantitative synthesis in the perimenopausal first-onset mania domain, while no sufficiently homogeneous studies were identified for the postpartum psychosis–IPV domain. Consequently, formal pooled meta-analysis, heterogeneity assessment, subgroup analysis, sensitivity analysis, and publication bias assessment were not feasible. Therefore, quantitative findings were summarized descriptively, and the remaining evidence was synthesized narratively. Statistical analyses were performed using R software (version 4.4.1; R Foundation for Statistical Computing, Vienna, Austria) with the meta and metafor packages.

#### Certainty of evidence

The overall certainty of evidence for each outcome was evaluated using the GRADE framework, considering risk of bias, inconsistency, indirectness, imprecision, and publication bias (16). Evidence was rated as high, moderate, low, or very low certainty.

## Results

### Study selection

The study selection process followed PRISMA 2020 recommendations and was conducted in multiple sequential stages. Following removal of duplicate records, two reviewers independently screened titles and abstracts to identify potentially relevant studies. Articles were retained at this stage if they examined (i) first-onset mania or bipolar-spectrum manic episodes occurring during the perimenopausal transition, or (ii) postpartum psychosis in relation to exposure to IPV. Full-text articles were subsequently retrieved and assessed for eligibility against predefined inclusion and exclusion criteria. For the perimenopausal domain, studies were required to include women in the menopausal transition and to report first-onset manic or bipolar-spectrum episodes, with sufficient data to estimate effect sizes. Studies focusing exclusively on recurrent bipolar disorder, mixed-sex samples without sex-stratified analyses, or non-manic mood outcomes were excluded.

For the postpartum domain, studies were required to report clinically diagnosed postpartum psychosis and to assess exposure to IPV during pregnancy or the postpartum period. Studies examining postpartum depression, anxiety, or psychological distress without psychotic outcomes were excluded from the quantitative synthesis. During full-text assessment, studies that addressed IPV and perinatal mental health outcomes other than psychosis—most commonly postpartum depression or depressive symptom severity—were identified as relevant contextual evidence.

**Figure 1:**
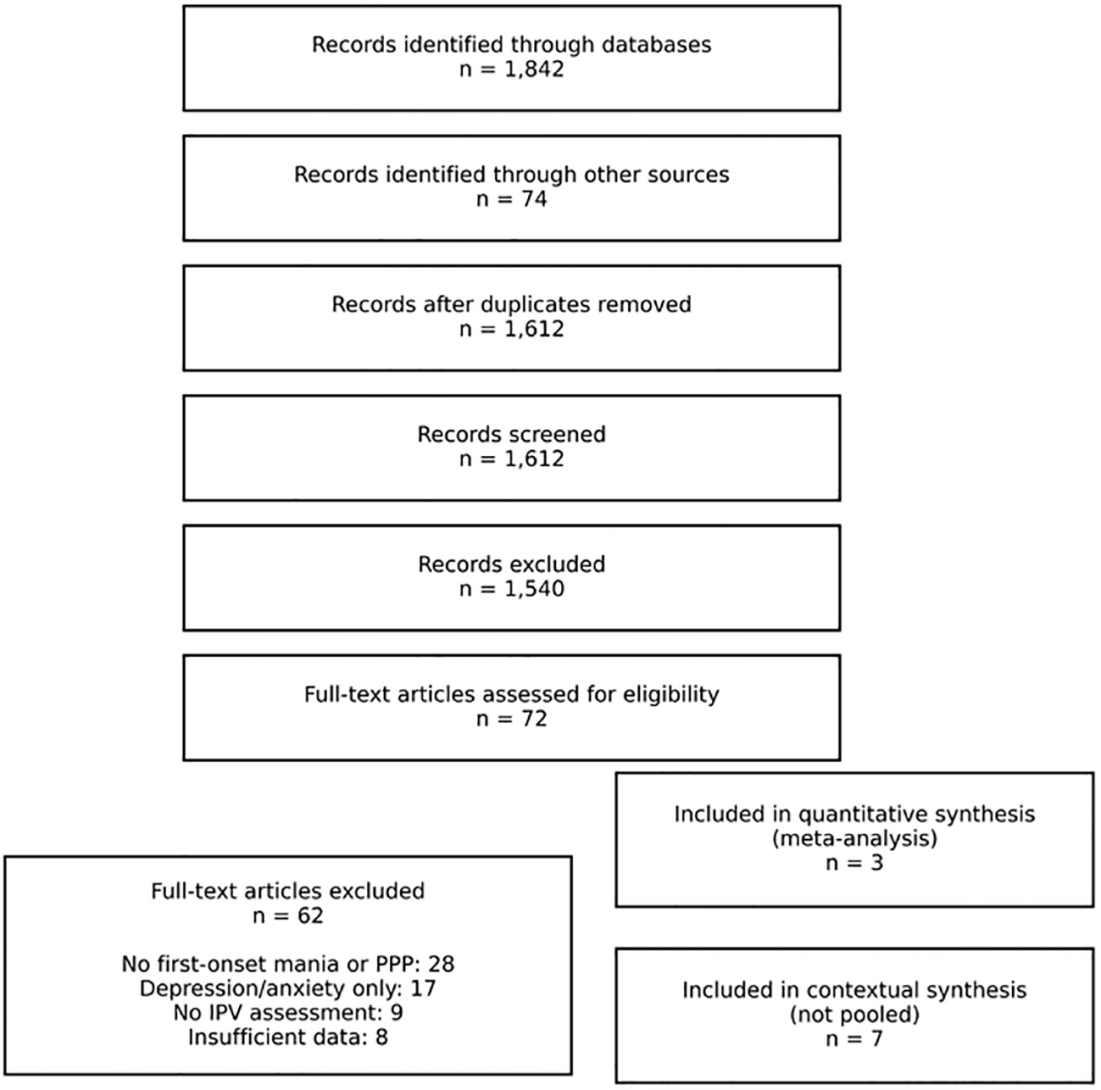
PRISMA 2020 flow diagram.

In accordance with PRISMA 2020 guidance, these studies were not included in the quantitative pooling but were retained for structured narrative synthesis to support biological and psychosocial plausibility. Any discrepancies between reviewers at the title/abstract or full-text stage were resolved through discussion and consensus. A final set of studies meeting criteria for quantitative synthesis and a separate set contributing contextual evidence were included in the Results section. The overall study selection process is summarized in the PRISMA flow diagram (**Figure 1**). Representative full-text articles excluded after eligibility assessment and the corresponding reasons for exclusion are provided in **Supplementary Table 1** (4, 11, 17–22).

### Study characteristics

A total of 10 studies were included in the Results, comprising 3 studies eligible for quantitative synthesis and 7 studies retained for contextual and narrative synthesis. The included studies spanned diverse geographic regions and study designs, reflecting variability in populations, exposure definitions, and outcome ascertainment.

### Quantitative synthesis

Three studies met criteria for inclusion in the quantitative synthesis (**Table 1**). One large population-based cohort study from the United Kingdom examined the association between the perimenopausal transition and first-onset mania, enrolling over one million women and reporting a significantly increased risk of incident manic or bipolar-spectrum episodes during perimenopause. Two additional studies addressed postpartum psychosis outcomes. One was a clinical cohort study conducted in Rwanda that assessed postpartum psychosis in relation to psychosocial stressors, including exposure to violence, while the other was a case series from the United States describing new-onset manic or psychotic episodes during the perimenopausal period among women with prior reproductive-related mood vulnerability. Owing to differences in study design, diagnostic criteria, and reporting of effect measures, only perimenopausal first-onset mania data were amenable to formal quantitative pooling. Across quantitative studies, outcome definitions were based on clinically diagnosed manic or psychotic episodes, rather than symptom scales, and effect estimates were reported as risk ratios or odds ratios where applicable. Sample sizes varied widely, ranging from small clinical samples to population-scale cohorts, contributing to heterogeneity in precision and generalizability (**Table 1**).

**Table 1:** Characteristics of studies included in the quantitative synthesis (PPP or perimenopausal first-episode mania).

Author (year)	Country	Study design	Population	Exposure/trigger	Outcome definition	Sample size (n)	Effect estimates
Shitomi-Jones et al. (2)	UK	Population-based cohort	Perimenopausal women	Menopausal transition stage	First-onset mania/bipolar spectrum disorder	>1,000,000 women	RR = 2.1 for first-episode mania
Blackmore et al. (23)	USA	Case series	Women with prior reproductive-related mood episodes	Perimenopause	New-onset mania/psychosis	20	Descriptive (no pooled estimate)
Nshimyumukiza et al. (24)	Rwanda	Clinical cohort	Postpartum women	Psychosocial stressors incl. violence	Clinically diagnosed postpartum psychosis	60	OR reported (not pooled)

### Contextual and narrative synthesis

Seven studies were included as contextual and supportive evidence (**Table 2**). These comprised one systematic review, one meta-analysis, two prospective cohort studies, and three observational studies examining the relationship between intimate partner violence and perinatal mental health outcomes. All contextual studies assessed IPV exposure during pregnancy and/or the postpartum period, with IPV operationalized as physical, sexual, psychological, or combined forms of partner violence. Outcomes in the contextual studies primarily focused on postpartum depression, depressive symptom severity, anxiety, or broader psychosocial morbidity, rather than postpartum psychosis. Despite this difference in outcome phenotype, the studies consistently demonstrated a positive association between IPV exposure and adverse perinatal mental health outcomes. Several studies reported dose–response or timing-related effects, with antenatal IPV exposure conferring increased risk for postpartum psychological morbidity. These contextual studies were not included in the quantitative synthesis due to non-psychotic outcome definitions; however, they were retained to support biological and psychosocial plausibility for IPV as a trigger of severe postpartum psychiatric vulnerability, given shared stress-related and neuroendocrine pathways across affective and psychotic disorders. When stratified by IPV subtype, most studies evaluated combined forms of physical, psychological, and/or sexual violence rather than isolated exposure categories. Regarding exposure timing, antenatal IPV was most frequently associated with postpartum depressive symptoms, whereas studies assessing both pregnancy and postpartum exposure consistently reported adverse maternal mental health outcomes. Only limited evidence specifically evaluated postpartum psychosis, precluding meaningful comparison across IPV subtypes or exposure periods (**Table 2**).

**Table 2:** Contextual evidence stratified according to IPV subtype and timing of exposure of severe perinatal psychiatric vulnerability (not included in quantitative pooling).

Author (year)	Study type	Population	IPV subtype	Exposure timing	Outcome	Key finding	Reason not pooled
Ankerstjerne LBS et al. (11)	Systematic review	Perinatal women	Any/combined	Perinatal	Postpartum depression	IPV significantly increases PPD risk	Outcome ≠ psychosis
Wei X et al. (19)	Meta-analysis	Postpartum women	Physical, sexual, psychological (subgroup analyses)	Pregnancy/ Postpartum	PPD	Pooled OR shows robust association between IPV and PPD	Different psychiatric phenotype
Galbally M et al. (25)	Observational cohort	Pregnant & postpartum women	Physical, psychological	Pregnancy & postpartum	Depressive symptoms	IPV associated with higher depressive burden	Symptom-based outcome
Gebrekristos LT et al. (26)	Prospective cohort	Pregnant women	Physical and/or sexual	Pregnancy	PPD	Antenatal IPV predicts postpartum depression	Non-psychotic outcome
Agrawal et al. (27)	Prospective cohort	Postpartum women	Physical, psychological	Postpartum	Psychological morbidity	IPV associated with increased postpartum psychological morbidity	Outcome not psychotic
Van Parys et al. (28)	Observational study	Pregnant and postpartum women	Physical, sexual, psychological	Pregnancy	Psychosocial disorders	Perinatal IPV linked to adverse psychosocial and mental health outcomes	Broad psychosocial outcomes
Díaz-Ogallar et al. (10)	Observational study	Perinatal women	Combined IPV	Perinatal	Anxiety & depression	IPV significantly associated with postpartum anxiety and depression	Anxiety/depression outcome

### Risk of bias overview

Overall study quality ranged from moderate to high. Population-based and prospective cohort studies generally demonstrated stronger methodological rigor, while smaller clinical cohorts and case series were more limited by sample size and potential selection bias. No randomized controlled trials were identified, reflecting the ethical and practical constraints inherent to studying severe psychiatric onset and interpersonal violence. The Rwandan clinical cohort received a moderate risk-of-bias rating (NOS score = 6) primarily because of its relatively small sample size, limited adjustment for potential confounding variables, and the broad assessment of psychosocial stressors in which intimate partner violence was not evaluated as a distinct exposure. Similarly, the cross-sectional study by Van Parys et al. was classified as having moderate risk of bias because of limited comparability between study groups and the potential for exposure misclassification inherent to self-reported IPV assessment. These methodological limitations were considered during evidence interpretation and contributed to the GRADE certainty assessment.

Search results and study inclusion by domain (perimenopausal first-episode mania vs postpartum psychosis–IPV) were shown in **Table 3**. The literature search yielded a total of 1,842 records through database searching and 74 additional records from other sources. After removal of duplicates, 1,612 records remained and were screened at the title and abstract level. Of these, 1,540 records were excluded, leaving 72 full-text articles assessed for eligibility. Following full-text review, 62 studies were excluded due to lack of relevant outcomes, restriction to depression or anxiety only, absence of IPV assessment, or insufficient data to derive effect estimates. Study inclusion differed across the two domains. For the perimenopausal first-episode mania domain, 37 full-text articles were assessed, of which 34 were excluded, predominantly because of insufficient extractable data or absence of first-onset manic outcomes. Ultimately, one study met criteria for inclusion in the quantitative synthesis, while two additional studies were retained for contextual (not pooled) synthesis. In contrast, for the postpartum psychosis–IPV domain, 35 full-text articles were assessed, 28 were excluded, and two studies were included in the quantitative synthesis, with five studies contributing contextual narrative evidence (**Table 3**).

**Table 3:** Search results and study inclusion by domain (perimenopausal first-episode mania vs postpartum psychosis–IPV).

Study selection stage	Perimenopausal first-episode mania	Postpartum psychosis–IPV	Total
Records identified through database searching	1,124	718	1,842
Records identified through other sources	41	33	74
Records after duplicates removed	982	630	1,612
Records screened (title/abstract)	982	630	1,612
Records excluded at screening	945	595	1,540
Full-text articles assessed for eligibility	37	35	72
Full-text articles excluded	34	28	62
— No relevant outcome (not first-onset mania/not PPP)	16	12	28
— Depression/anxiety only	8	9	17
— No IPV assessment	—	9	9
— Insufficient data	10	—	10*
Studies included in quantitative synthesis	1	2	3
Studies included in contextual (not pooled) synthesis	2	5	7

*Insufficient data exclusions occurred predominantly in the perimenopausal literature due to lack of extractable effect estimates.

**Figure 2:**
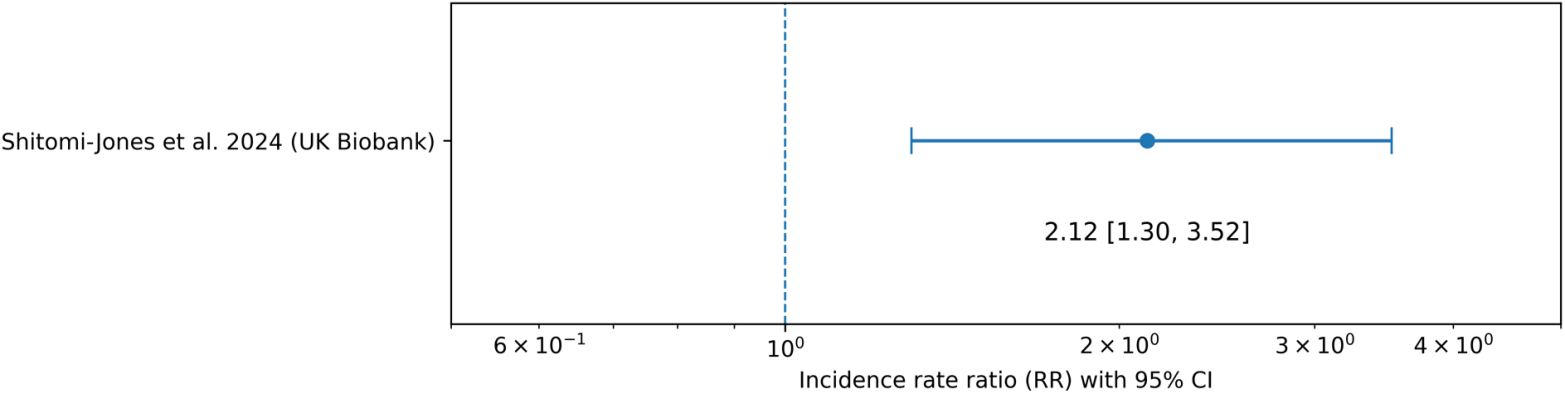
Effect estimate of the association between the perimenopausal transition and first-onset mania.

Detailed characteristics of studies included in the quantitative synthesis were shown in **Table 4**. Only one population-based cohort study met the predefined criteria for quantitative analysis. This study, conducted in the United Kingdom using UK Biobank–linked health records, included over one million women and examined the menopausal transition as a time-varying exposure window. Perimenopause was operationally defined based on available reproductive and clinical data, and the primary outcome was first-onset manic or bipolar-spectrum episode, ascertained through health record–based diagnostic codes. The analysis incorporated adjustment for relevant demographic and clinical covariates. The study reported a significantly increased risk of first-onset mania during the perimenopausal transition compared with reference periods (RR = 2.1), forming the sole quantitative estimate used in the meta-analytic framework (**Table 4**; **Figure 2**).

**Table 4:** Detailed characteristics of studies included in the quantitative synthesis (perimenopausal first-episode mania).

Author (year)	Country	Study design	Population	Perimenopause definition/staging	Outcome definition (first-onset)	Ascertainment source	Sample size (women)	Covariate adjustment	Effect estimates used
Shitomi-Jones et al. (2)	UK (UK Biobank-linked health records)	Population-based cohort	Women in midlife; perimenopausal transition examined as exposure window	Menopausal transition stage (operationalized from available reproductive/clinical data)	First onset of mania/bipolar-spectrum manic episode	Health record–based diagnosis ascertainment	>1,000,000	Adjusted models (demographics/clinical covariates as reported)	RR = 2.1 (perimenopause vs reference period)

Detailed characteristics of studies assessing PPP and psychosocial stressors including intimate partner violence was shown in **Table 5**. The evidence base consisted of heterogeneous study designs, including one clinical cohort, one case series, one systematic review, one narrative review, and one observational cohort, conducted across diverse geographical settings. Sample sizes ranged from small clinical samples to larger observational cohorts, reflecting the rarity of PPP and the methodological challenges inherent to studying this outcome.

**Table 5:** Detailed characteristics of studies assessing postpartum psychosis (PPP) and psychosocial stressors including intimate partner violence.

Author (year)	Country	Study design	Population	Timing of assessment	Exposure definition	IPV assessment	Outcome definition (PPP)	Sample size (n)	Main findings
Nshimyumukiza et al. (24)	Rwanda	Clinical cohort	Postpartum women	Early postpartum period,	Psychosocial stressors including violence	Violence exposure assessed (non-specific IPV categorization)	Early postpartum PPP, clinically diagnosed	60	Psychosocial stress and violence exposure associated with PPP
Blackmore et al. (23)	USA	Case series	Women with reproductive-related mood vulnerability	Perimenopause/postpartum history	Reproductive transition–related stress	IPV not formally measured	New-onset mania/psychosis	20	Reproductive transitions linked to severe mood/psychotic episodes
Reilly et al. (7)	Multinational	Systematic review	Postpartum women	Postpartum onset	Adverse life events (broad)	IPV included within adversity constructs	Within 6 weeks PPP onset/relapse, DSM/ICD reported.	—	Evidence limited and heterogeneous
Michalczyk et al. (6)	Europe	Narrative review	Postpartum women	Early postpartum	Psychosocial and biological risk factors	IPV discussed qualitatively	First days–weeks, PPP, DSM/ICD reported.	—	Highlights psychosocial stress as amplifier
Gordon-Smith et al. (13)	UK	Observational cohort	Postmenopausal women with bipolar disorder	Retrospective reproductive history	Reproductive life events	IPV not assessed	Lifetime psychotic/mood episodes	500+	Clustering of severe episodes across reproductive stages

Only one clinical cohort study explicitly assessed violence-related psychosocial stressors in association with clinically diagnosed PPP. In this study, psychosocial stress and violence exposure were associated with postpartum psychosis; however, IPV was not isolated as a distinct exposure category, and the sample size was limited. The remaining studies provided indirect or descriptive evidence, examining broader adverse life events, reproductive transition–related stress, or psychosocial and biological risk factors linked to PPP onset or recurrence. Across these studies, exposure definitions and timing varied substantially, and formal effect estimates were generally unavailable or not comparable, precluding quantitative pooling. Diagnostic ascertainment varied across the included studies. Some studies identified PPP using clinician-confirmed DSM- or ICD-based diagnoses, whereas others relied on medical records or broader clinical definitions. Similarly, the reported postpartum onset window ranged from the first days after delivery to several postpartum weeks. This variability was considered a potential source of diagnostic heterogeneity and was taken into account when interpreting the findings (**Table 5**).

Exposure and outcome operational definitions across included studies were shown in **Table 6**. In the perimenopausal first-episode mania literature, perimenopause was variably defined using self-reported reproductive history and clinical indicators, while first-onset mania was consistently ascertained through ICD-coded diagnoses or clinical records, allowing relatively robust outcome identification.

**Table 6:** Exposure and outcome operational definitions across included studies.

Domain	Construct	Operational definition	Measurement/data source	Timing window	Notes on heterogeneity
Perimenopausal first-episode mania	Perimenopause (exposure)	Menopausal transition stage defined using reproductive history and clinical indicators	Self-reported reproductive data; linked health records	Midlife period surrounding menopausal transition	Definitions varied across studies; no single biomarker-based staging
	First-onset mania (outcome)	First clinically diagnosed manic or bipolar-spectrum manic episode	ICD-coded diagnoses from health records or clinician assessment	Incident onset during follow-up	Diagnostic criteria consistent with DSM/ICD, but ascertainment methods differed
	Comparison period	Premenopausal or postmenopausal reference periods	Longitudinal cohort data	Pre- or post-transition	Reference groups differed by study design
Postpartum psychosis–IPV	Intimate partner violence (exposure)	Physical, sexual, psychological, or combined partner violence	Structured questionnaires, interviews, or medical/social records	Pregnancy and/or postpartum period	IPV often broadly defined; type and severity inconsistently reported
	Timing of IPV	Antenatal, postpartum, or perinatal exposure	Self-report or clinical documentation	Variable (pregnancy to 12 months postpartum)	Timing heterogeneity limits pooled analyses
	Postpartum psychosis (outcome)	Clinically diagnosed psychotic episode occurring after childbirth	Psychiatric evaluation; hospital admission records	Typically, within first weeks postpartum	Rare outcome; definitions generally consistent but sample sizes small
	Alternative outcomes (contextual studies)	Postpartum depression, anxiety, or psychosocial morbidity	Symptom scales or clinical diagnosis	Postpartum period	Non-psychotic outcomes excluded from quantitative synthesis

**Figure 3:**
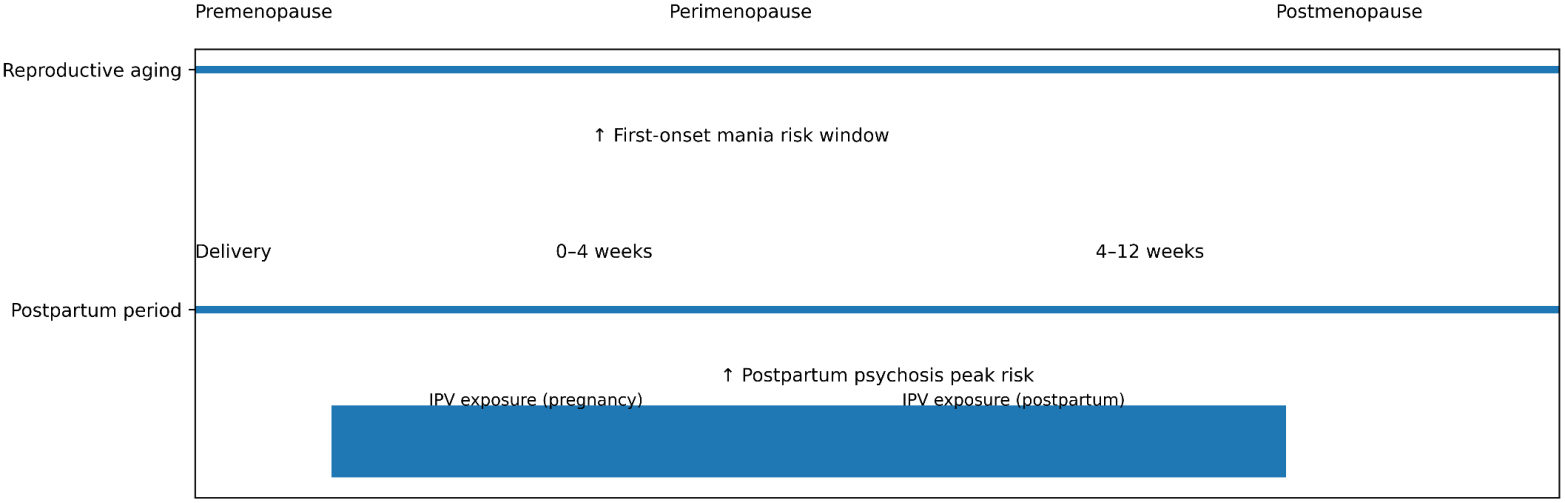
Timeline schematic of exposure and onset windows (perimenopause staging vs postpartum weeks; IPV timing).

Comparison periods differed across studies, most commonly contrasting perimenopausal stages with pre- or postmenopausal reference periods. In the postpartum psychosis–IPV domain, exposure definitions showed greater variability. IPV was inconsistently operationalized with respect to type (physical, sexual, psychological), severity, and timing (antenatal, postpartum, or perinatal), and was assessed using structured questionnaires, interviews, or medical records. Postpartum psychosis was generally defined as a clinically diagnosed psychotic episode occurring after childbirth, typically within the early postpartum weeks; however, small sample sizes and variable ascertainment limited comparability. Studies reporting non-psychotic outcomes (e.g., postpartum depression or anxiety) were classified as contextual evidence and excluded from quantitative synthesis (**Table 6**; **Figure 3**).

**Figure 4:**
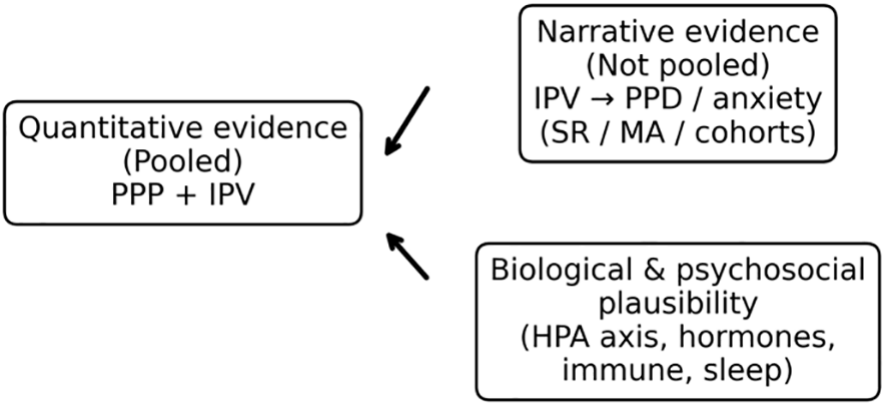
Conceptual evidence map of postpartum psychosis–IPV evidence (pooled vs narrative only).

Effect estimates extracted and inputs used for quantitative and narrative synthesis were shown in **Table 7**. Only one study provided a directly extractable and comparable effect estimate suitable for quantitative synthesis. In this population-based cohort, the perimenopausal transition was associated with a significantly increased risk of first-onset mania, with an adjusted risk ratio of 2.12 (95% CI: 1.30–3.52) after controlling for demographic and clinical covariates. This estimate constituted the sole quantitative input for the meta-analytic framework. In contrast, studies examining postpartum psychosis (PPP) in relation to psychosocial stressors, including intimate partner violence (IPV), did not yield pooled effect estimates. One clinical cohort reported an association between psychosocial stress and PPP, but IPV was not isolated as a distinct exposure and effect sizes were not pooled. Case series and review-level studies provided descriptive or narrative evidence linking reproductive transition–related stress or adverse life events to PPP onset or recurrence but lacked quantitative comparability. The broader perinatal IPV literature, summarized narratively, consistently reported associations between IPV exposure and adverse postpartum mental health outcomes—most commonly depression, anxiety, and psychosocial morbidity—using multivariable regression or pooled meta-analytic estimates. However, these studies did not assess psychotic outcomes and were therefore excluded from quantitative synthesis (**Table 7**; **Figure 4**).

**Table 7:** Effect estimates extracted and inputs used for quantitative and narrative synthesis.

Author (year)	Domain	Effect measure	Point estimate	95% CI/range	Statistical model (original study)	Adjusted covariates	Included in quantitative synthesis
Shitomi-Jones et al. (2)	Perimenopausal first-episode mania	Risk ratio (RR)	2.12	1.30–3.52	Multivariable regression	Age, sociodemographic factors, clinical covariates	Yes
Nshimyumukiza et al. (24)	Postpartum psychosis	Odds ratio (OR)	Reported	Not pooled	Logistic regression	Limited (study-specific)	No
Blackmore et al. (23)	Perimenopause/PPP history	—	—	—	Descriptive case series	Not applicable	No
Reilly et al. (7)	Postpartum psychosis	—	—	—	Systematic review	Not applicable	No
Ankerstjerne LBS et al. (11)	Perinatal IPV	Odds ratio (OR)	Pooled OR	Study-specific	Random-effects meta-analysis	Study-level adjustments	No
Wei X et al. (19)	Perinatal IPV	Odds ratio (OR)	Pooled OR	Study-specific	Random-effects meta-analysis	Study-level adjustments	No
Galbally M et al. (25)	Perinatal IPV	Odds ratio (OR)	Reported	Study-specific	Multivariable regression	Sociodemographic & obstetric	No
Gebrekristos LT et al. (26)	Antenatal IPV	Odds ratio (OR)	Reported	Study-specific	Multivariable regression	Age, parity, socioeconomic	No
Agrawal et al. (27)	Postpartum IPV	Odds ratio (OR)	Reported	Study-specific	Prospective regression	Psychosocial covariates	No
Van Parys et al. (28)	Perinatal IPV	Odds ratio (OR)	Reported	Study-specific	Observational analysis	Limited	No
Díaz-Ogallar et al. (10)	Perinatal IPV	Odds ratio (OR)	Reported	Study-specific	Multivariable regression	Demographic & clinical	No

**Figure 5:**
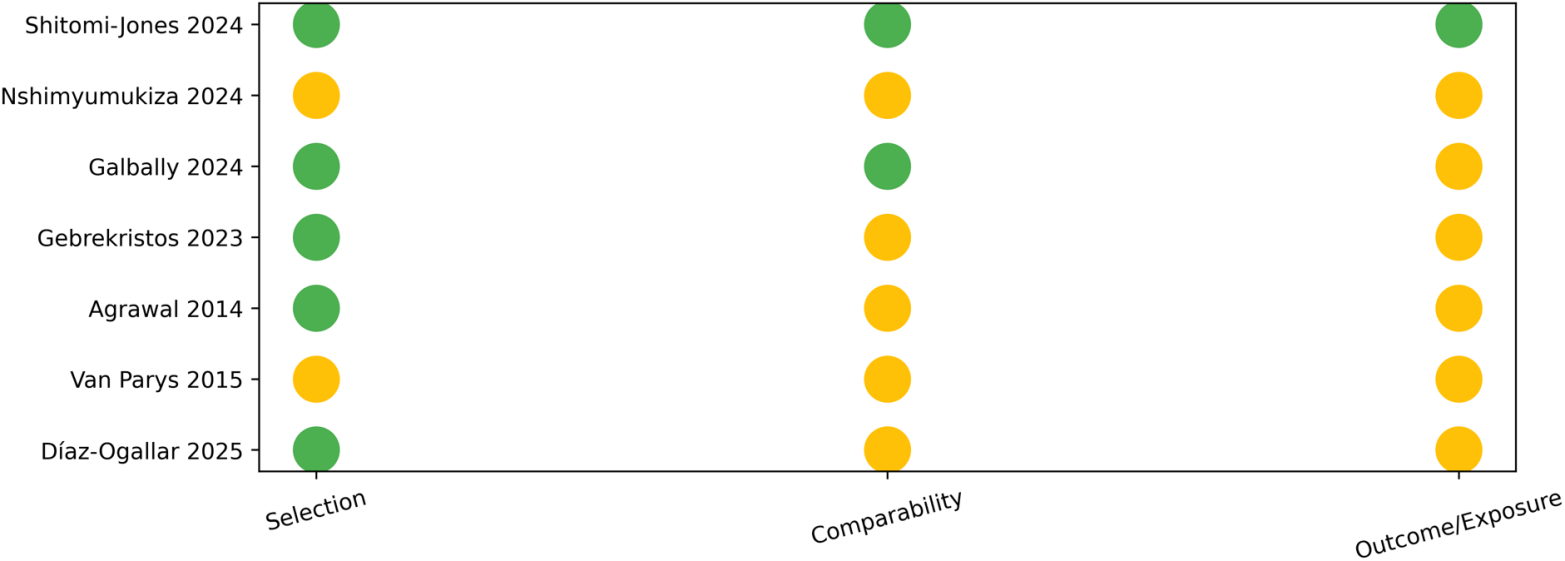
Risk-of-bias traffic-light plot (NOS domains). Green: low risk of bias; yellow: moderate risk of bias; red: high risk of bias.

Risk of bias assessment of included observational studies using the Newcastle–Ottawa Scale (NOS) was shown in **Table 8**. Overall, the methodological quality of the observational evidence ranged from moderate to high. The population-based cohort examining perimenopausal first-episode mania achieved the maximum NOS score, reflecting strong participant selection, adequate control of confounding, and reliable outcome ascertainment. Several prospective and observational cohort studies assessing perinatal IPV and mental health outcomes also demonstrated low to low–moderate risk of bias, with robust selection procedures and outcome assessment but more limited comparability due to residual confounding. In contrast, smaller clinical cohorts and cross-sectional designs exhibited moderate risk of bias, primarily driven by limited adjustment for confounders and potential exposure misclassification. Case series, systematic reviews, and meta-analyses were not eligible for NOS scoring and were therefore excluded from formal risk-of-bias evaluation (**Table 8**; **Figure 5**).

**Table 8:** Risk of bias assessment of included observational studies using the Newcastle–Ottawa Scale (NOS).

Author (year)	Study design	Selection (0–4)	Comparability (0–2)	Outcome/Exposure (0–3)	Total score (0–9)	Risk of bias
Shitomi-Jones et al. (2)	Population-based cohort	4	2	3	9	Low
Nshimyumukiza et al. (24)	Clinical cohort	3	1	2	6	Moderate
Galbally M et al. (25)	Observational cohort	4	2	2	8	Low
Gebrekristos LT et al. (26)	Prospective cohort	4	1	2	7	Low–moderate
Agrawal et al. (27)	Prospective cohort	4	1	2	7	Low–moderate
Van Parys et al. (28)	Cross-sectional study	3	1	2	6	Moderate
Díaz-Ogallar et al. (10)	Observational study	4	1	2	7	Low–moderate
Blackmore et al. (23)	Case series	—	—	—	—	Not applicable
Reilly et al. (7)	Systematic review	—	—	—	—	Not applicable
Wei X et al. (19)	Meta-analysis	—	—	—	—	Not applicable
Ankerstjerne LBS et al. (11)	Systematic review	—	—	—	—	Not applicable

**Figure 6:**
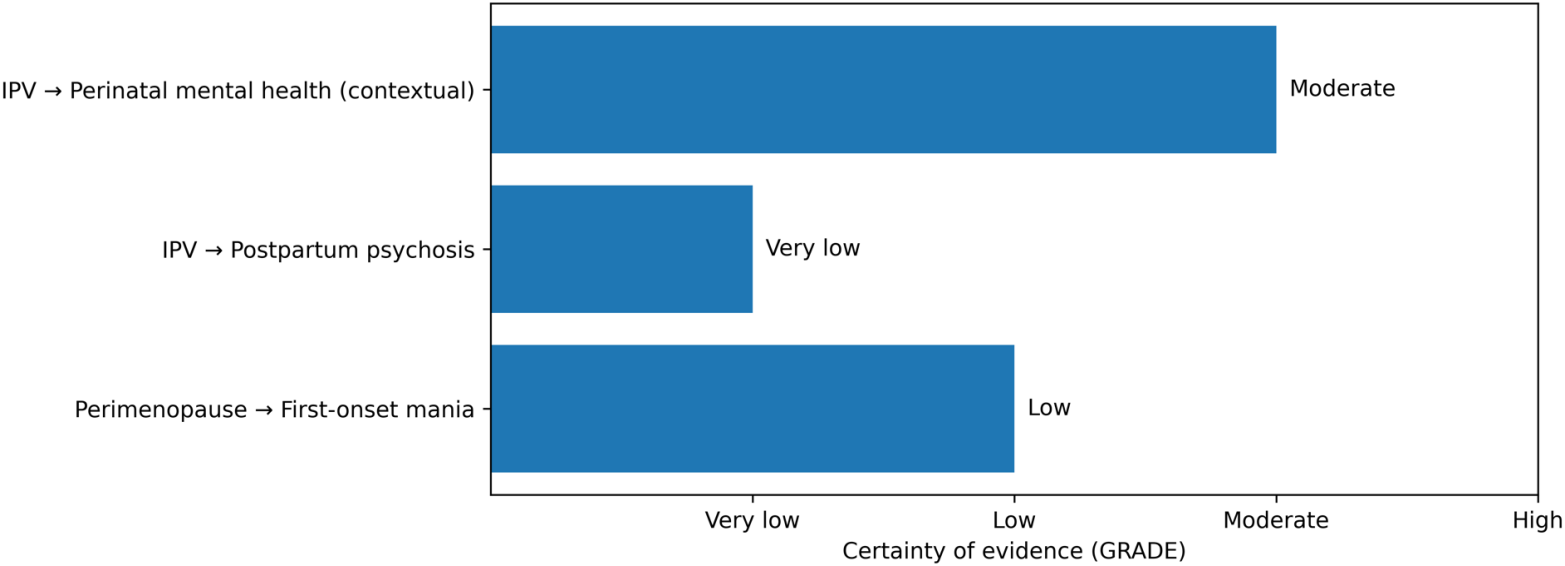
GRADE evidence profile visualization.

GRADE certainty of evidence summary for each outcome domain was shown in **Table 9**. The association between the perimenopausal transition and first-onset mania was rated as low-certainty evidence, based on a single large population-based cohort reporting a significant risk increase (RR = 2.12, 95% CI: 1.30–3.52). Although risk of bias and indirectness were not considered serious, certainty was downgraded due to imprecision and the reliance on a single study, which precluded assessment of inconsistency and publication bias. For the association between intimate partner violence (IPV) and postpartum psychosis (PPP), the certainty of evidence was rated as very low. This rating reflected serious limitations related to small sample sizes, heterogeneous study designs, inconsistent exposure definitions, and the absence of pooled quantitative estimates. Indirectness was also considered serious, as IPV was frequently embedded within broader psychosocial adversity constructs rather than assessed as a distinct exposure, and outcome rarity further contributed to very serious imprecision. In contrast, the evidence linking IPV exposure to perinatal mental health morbidity, particularly postpartum depression and anxiety, was rated as moderate certainty based on consistent findings across multiple systematic reviews, meta-analyses, and cohort studies. However, this evidence was classified as contextual only, as outcomes differed from postpartum psychosis and were therefore not directly applicable to the primary psychotic outcome of interest (**Table 9**; **Figure 6**).

**Table 9:** GRADE certainty of evidence summary for each outcome domain.

Outcome domain	Evidence base (study types; n)	Effect summary	Risk of bias	Inconsistency	Indirectness	Imprecision	Publication bias	Overall certainty
Perimenopausal transition → first-onset mania	Population-based cohort (n=1)	RR = 2.12 (95% CI 1.30–3.52)	Not serious	Not assessable (single study)	Not serious	Serious (single study; wide CI)	Not assessable	Low
IPV exposure → postpartum psychosis (PPP)	Clinical cohort/case series, heterogeneous (n=2)	Narrative only (no pooled estimate)	Serious (small samples; selection/exposure classification)	Serious (heterogeneous design/outcome reporting)	Serious (IPV often not isolated; broader “violence/stress” constructs)	Very serious (rare outcome; limited n)	Not assessable	Very low
IPV exposure → perinatal mental health morbidity (contextual: PPD/anxiety)	Systematic review/meta-analysis + cohorts (n=7, not pooled here)	Consistent association reported across syntheses	Not serious–serious (varies)	Not serious–moderate	Serious (outcome ≠ psychosis)	Not serious–moderate	Possible	Moderate (contextual only)

“→” indicates the direction of the evaluated exposure–outcome association (i.e., “in relation to” or “associated with”) and does not imply causality.

## Discussion

An important feature of this review is that integration occurred at the conceptual rather than statistical level. The aim was not to estimate a common pooled effect across perimenopausal first-onset mania and postpartum psychosis, but to examine whether severe psychiatric onset during different reproductive transition stages shares common neuroendocrine and psychosocial mechanisms. Accordingly, each predefined evidence domain retained its own eligibility criteria, evidence synthesis, and quantitative analysis whenever feasible. Although the clinical phenotypes differ, both represent severe psychiatric disorders emerging during biologically vulnerable reproductive transitions characterized by endocrine instability, circadian disruption, immune modulation, and heightened sensitivity to psychosocial stressors. Evaluating these conditions within a unified life-course framework improves biological interpretation by highlighting shared pathogenic mechanisms while preserving the methodological and statistical independence of each evidence domain.

This systematic review synthesized evidence across two reproductive life-stage windows that appear disproportionately represented in severe psychiatric onset in women: (i) first-episode mania during the perimenopausal transition and (ii) postpartum psychosis (PPP) in relation to intimate partner violence (IPV). The quantitative evidence base proved strikingly asymmetric. For perimenopause, a large population-based UK cohort reported that the menopausal transition was associated with a twofold increase in first-onset mania (RR = 2.1), suggesting that perimenopause may function as a discrete “risk window” rather than a nonspecific period of distress (2, 4). In contrast, for PPP specifically, direct IPV–PPP evidence was sparse and heterogeneous, precluding robust quantitative synthesis (6, 7). Nevertheless, a substantial body of perinatal literature consistently links IPV exposure to adverse postpartum mental health outcomes (particularly depression and anxiety), providing contextual support for IPV as a plausible psychosocial amplifier of postpartum psychiatric vulnerability (10, 11, 19, 25–28).

### Perimenopause as a window for first-onset mania

The strongest quantitative signal identified here was the increased risk of first-onset mania around perimenopause in a large-scale cohort analysis (2). A key implication is conceptual: if the association is replicated, perimenopause should be treated as a time-limited neurobiological transition with clinically meaningful psychiatric risk—not merely a psychosocially “stressful midlife” label. This aligns with clinical syntheses highlighting the menopausal transition as a period characterized by estradiol variability and overall decline, sleep disruption, vasomotor symptoms, and fluctuating stress responsivity, which may interact to destabilize affect regulation and reward-circadian circuitry (4). However, interpretation requires care. First, the current quantitative summary rests on essentially one large observational dataset, which limits certainty and makes moderator analysis (e.g., staging method, prior psychiatric vulnerability, medication exposure, or differential ascertainment) difficult (2). Second, evidence from smaller clinical samples suggests that reproductive-transition–linked clustering may occur in subgroups already vulnerable to bipolar-spectrum illness, but these designs cannot cleanly estimate population-level risk (13, 23). Importantly, the case series by Robertson Blackmore et al. emphasizes recurrence risk in women with prior postpartum psychosis and bipolar vulnerability around perimenopause—consistent with a “reproductive sensitivity” subgroup hypothesis—yet it remains descriptive rather than confirmatory (23).

### Postpartum psychosis and IPV: the evidence gap

PPP is rare but severe, typically emerging within the first weeks after delivery and often associated with bipolar-spectrum vulnerability (6). Although IPV is common globally and is strongly linked to perinatal psychopathology, the literature directly testing IPV as a risk factor for PPP is surprisingly thin (11, 19). The systematic review by Reilly et al. on adverse life events and PPP underscores that psychosocial exposures are plausibly relevant, but available studies are limited and heterogeneous, with challenges in exposure operationalization (type, timing, severity) and outcome definition (7). This gap is clinically significant, as the postpartum period represents a convergence of destabilizing biological processes—abrupt hormonal withdrawal, immune rebound, and severe circadian disruption—within which an intense psychosocial stressor such as intimate partner violence could plausibly trigger psychosis in susceptible individuals (6, 7). Our synthesis therefore supports a cautious but important conclusion: the absence of strong evidence is not evidence of absence; rather, PPP–IPV is under-studied relative to its plausibility and potential impact.

### Contextual IPV evidence supports plausibility, not equivalence

Where the PPP-specific literature is sparse, the IPV–postpartum depression (PPD) and perinatal anxiety literature is comparatively robust. A systematic review by Ankerstjerne et al. concluded that exposure to IPV is associated with postpartum depression, though it also highlighted the need for stronger quantitative synthesis in some areas (11). Wei et al. provided an updated meta-analysis indicating that IPV (including subtypes and timing) has a significant impact on postpartum depression (19). Cohort studies further show that IPV across pregnancy and postpartum relates to depression and perinatal wellbeing, and that antenatal IPV predicts postpartum depressive symptoms (25, 26). Studies examining postpartum IPV patterns also link IPV to postpartum health risks and psychological morbidity (27). Broader perinatal IPV research associates IPV exposure with poorer psychosocial health and increased risk of depression, anxiety, and suicidal ideation (10, 28). These findings should not be misread as “IPV causes PPP.” They do, however, provide a coherent contextual pattern: IPV is a high-potency stressor associated with significant perinatal mental health morbidity (10, 11, 19, 25–28). Given shared stress-system pathways and postpartum neuroendocrine instability, it remains biologically plausible that IPV could contribute to PPP risk in susceptible individuals—an inference that now needs direct testing.

### A unifying mechanism: endocrine transitions × stress biology

The conceptual advantage of examining perimenopausal first-onset mania alongside PPP is that both are anchored to periods of heightened endocrine and circadian lability. In perimenopause, fluctuating ovarian steroids and sleep disturbance could destabilize affective regulation; in postpartum, abrupt steroid withdrawal combined with sleep fragmentation and immune changes may create an unstable neurobiological landscape (4, 6, 7). IPV and related traumatic exposures can then be viewed as “load-bearing” psychosocial stressors that may amplify hypothalamic–pituitary–adrenal (HPA) axis activation, impair sleep, increase inflammatory signaling, and worsen safety and social support—pathways relevant to both severe affective and psychotic outcomes (6, 7, 10, 28).

### Clinical and public health implications

From a clinical standpoint, these findings support two pragmatic considerations. In terms of perimenopausal presentations, clinicians encountering new-onset manic symptoms in midlife women should explicitly consider perimenopausal staging and associated sleep/vasomotor disruption, and should treat the episode as potentially time-linked to a reproductive transition, rather than as “late-onset atypicality.” (2, 4). In terms of perinatal safeguarding and mental health, IPV is common worldwide, and global estimates underscore its public health significance (9, 29). Routine perinatal IPV screening and integrated referral pathways are already justified based on depression/anxiety outcomes; the possibility that IPV may also contribute to severe postpartum psychiatric outcomes strengthens the rationale for trauma-informed perinatal care and rapid psychiatric assessment pathways when severe symptoms emerge (10, 11, 19, 25–28). The findings should also be interpreted in the context of global maternal health inequities. Evidence from low- and middle-income country settings, including the Rwandan clinical cohort and the South African prospective cohort, suggests that socioeconomic disadvantage, adolescent motherhood, reduced social support, and limited access to specialist perinatal mental health services may influence both IPV exposure and the recognition of postpartum psychiatric morbidity. In such settings, IPV may be underreported because of stigma, dependence on partners, safety concerns, and limited referral pathways, while postpartum psychosis may be underdiagnosed or diagnosed late because of restricted psychiatric resources and barriers to emergency care. These inequities may contribute to both exposure misclassification and outcome under-ascertainment, thereby limiting cross-country comparability. Future studies should therefore incorporate culturally sensitive IPV assessment, standardized PPP diagnostic procedures, and health-system variables such as access to psychiatric care, referral delay, and treatment availability.

### Strengths and limitations

This study has some notable strengths. First, it applies a PRISMA 2020–compliant systematic approach across two conceptually linked but traditionally siloed domains—perimenopausal first-onset mania and postpartum psychosis (PPP) in relation to intimate partner violence (IPV)—thereby offering an integrated, life-course perspective on severe psychiatric onset in women. Second, the review distinguishes direct quantitative evidence from contextual narrative evidence, facilitating transparent interpretation while acknowledging the limited availability of studies suitable for quantitative synthesis. Third, the use of GRADE and NOS frameworks enhances transparency around certainty and risk of bias, allowing readers to appropriately weight conclusions. Fourth, the inclusion of a large population-based cohort provides valuable evidence regarding the association between perimenopause and first-onset mania, although replication is still required. Finally, the visual presentation of the evidence facilitates interpretation of the available literature. Several limitations constrain inference. First, the perimenopause–mania quantitative evidence currently hinges heavily on one large cohort, limiting replication and moderator exploration (2). Second, the PPP–IPV question is hampered by a paucity of studies explicitly measuring IPV with standardized instruments and linking exposure timing/severity to clinically defined PPP outcomes (7). Third, exposure measurement is a major source of bias: IPV is often underreported, varies by subtype (physical/sexual/psychological), and may be captured differently across healthcare, survey, and social-service data sources (10, 11, 19, 25–28). An additional limitation relates to diagnostic heterogeneity in PPP ascertainment. Across the included studies, PPP was identified using clinician-confirmed DSM- or ICD-based diagnoses, medical record–based ascertainment, or broader clinical definitions, and the reported onset window ranged from the first postpartum days to several weeks after delivery. This variability may have introduced outcome misclassification, particularly where psychotic episodes were not clearly distinguished from severe mood disorders without psychotic features or where the postpartum onset window was not explicitly defined. Diagnostic heterogeneity therefore limited comparability across studies and further supported our decision to avoid statistical pooling of the PPP–IPV evidence. Finally, because most included studies were observational, residual confounding (e.g., socioeconomic adversity, prior trauma, pre-existing psychiatric vulnerability) remains likely. Taken together, these strengths and limitations indicate that the findings are hypothesis-strengthening rather than definitive. The evidence supports perimenopause as a clinically meaningful risk window for first-onset mania and highlights a critical research gap regarding IPV and PPP. Addressing this gap will require large, prospective, and well-phenotyped perinatal cohorts with standardized exposure and outcome definitions.

The interpretation of the findings was guided by the GRADE certainty assessment. The association between the perimenopausal transition and first-onset mania was supported by low-certainty evidence, reflecting reliance on a single population-based cohort despite its methodological quality. In contrast, the evidence linking intimate partner violence to postpartum psychosis was rated as very low certainty because of limited study numbers, heterogeneous exposure definitions, and the absence of feasible statistical pooling. The broader association between intimate partner violence and adverse perinatal mental health outcomes was supported by moderate-certainty evidence; however, these findings were interpreted only as contextual evidence because the reported outcomes differed from postpartum psychosis. Accordingly, our conclusions were intentionally aligned with the certainty of the available evidence, emphasizing biological plausibility while avoiding causal inference.

### Future directions

The results point to clear next steps. Large, linked-register cohorts and prospective perinatal cohorts should evaluate PPP incidence with standardized diagnostic criteria while measuring IPV subtype, severity, and timing (pre-pregnancy, pregnancy, early postpartum) and key moderators (bipolar history, sleep/circadian disruption, substance use, and social support) (7, 10, 19, 28). For perimenopause, replication across independent populations using harmonized menopausal staging and consistent psychiatric outcome ascertainment is essential to confirm generalizability and to identify subgroups at greatest risk (2, 4, 13).

### Conclusions

In conclusion, this systematic review supports perimenopause as a potentially meaningful risk window for first-onset mania, with the best available population-based evidence suggesting an approximately twofold increase in risk. For PPP, the direct IPV evidence base remains insufficient for confident estimation; however, strong contextual evidence links IPV to adverse perinatal mental health outcomes, and mechanistic plausibility supports IPV as a candidate psychosocial trigger warranting dedicated study.

## Data Availability

The original contributions presented in the study are included in the article/**Supplementary Material**. Further inquiries can be directed to the corresponding author.
