# Supplementary material for: Neuroendocrine and psychosocial triggers of severe psychiatric onset in women: a systematic review with domain-specific quantitative and narrative synthesis
Source: Front Psychiatry. 2026 Jul 29;17:1889528. doi: 10.3389/fpsyt.2026.1889528 (PMC13461523; doi:10.3389/fpsyt.2026.1889528)
Supplement: Supplementary file 1 [file Table1.docx]

**Supplementary Table S1.** Representative full-text articles excluded after eligibility assessment and reasons for exclusion 4,11,17-22].

| **Study** | **Domain** | **Primary reason for exclusion** |
| --- | --- | --- |
| Marsh et al. | Perimenopausal first-onset mania | Included women with established bipolar disorder; first-onset mania was not evaluated. |
| Musial et al. | Perimenopausal first-onset mania | Narrative/clinical review; no original quantitative data suitable for effect estimation. |
| Howard et al. | PPP–IPV | Examined domestic violence and broad perinatal mental disorders; postpartum psychosis was not isolated as a primary outcome. |
| Wei et al. | PPP–IPV | Meta-analysis of IPV and postpartum depression; psychotic outcomes were not assessed. |
| Ankerstjerne et al. | PPP–IPV | Systematic review focused on IPV and postpartum depression; outcome did not meet PPP eligibility criteria. |
| Ashenafi et al. | PPP–IPV | Assessed IPV during pregnancy and postpartum depression; clinically diagnosed postpartum psychosis was not reported. |
| Navarrete et al. | PPP–IPV | Evaluated perinatal depression/anxiety in relation to IPV; psychotic outcomes were absent. |
| Kenny/Velonis et al. | PPP–IPV | Examined prenatal/postpartum partner violence and maternal health outcomes; PPP was not assessed. |
